# Long-Term Motor Cortical Electrical Stimulation Ameliorates 6-Hydroxydopamine-Induced Motor Dysfunctions and Exerts Neuroprotective Effects in a Rat Model of Parkinson’s Disease

**DOI:** 10.3389/fnagi.2022.848380

**Published:** 2022-02-16

**Authors:** Chi-Wei Kuo, Ming-Yuan Chang, Ming-Yi Chou, Chien-Yuan Pan, Chih-Wei Peng, Hui-Chiun Tseng, Tsu-Yi Jen, Xiao-Kuo He, Hui-Hua Liu, Thi Xuan Dieu Nguyen, Pi-Kai Chang, Tsung-Hsun Hsieh

**Affiliations:** ^1^School of Physical Therapy and Graduate Institute of Rehabilitation Science, Chang Gung University, Taoyuan City, Taiwan; ^2^Department of Life Science, National Taiwan University, Taipei City, Taiwan; ^3^Division of Neurosurgery, Department of Surgery, Min-Sheng General Hospital, Taoyuan City, Taiwan; ^4^Department of Early Childhood and Family Educare, Chung Chou University of Science and Technology, Yuanlin City, Taiwan; ^5^Graduate Institute of Neural Regenerative Medicine, College of Medical Science and Technology, Taipei Medical University, Taipei City, Taiwan; ^6^School of Biomedical Engineering, College of Biomedical Engineering, Taipei Medical University, Taipei City, Taiwan; ^7^Department of Psychology, National Taiwan University, Taipei City, Taiwan; ^8^Department of Rehabilitation Medicine, The Fifth Hospital of Xiamen, Xiamen, China; ^9^Department of Rehabilitation Medicine, Sun Yat-sen Memorial Hospital, Sun Yat-sen University, Guangzhou, China; ^10^Neuroscience Research Center, Chang Gung Memorial Hospital, Taoyuan City, Taiwan; ^11^Healthy Aging Research Center, Chang Gung University, Taoyuan City, Taiwan

**Keywords:** cortical electrical stimulation, Parkinson’s disease, gait, locomotor function, neuroprotection, 6-OHDA, rats

## Abstract

**Objective:**

Cortical electrical stimulation (CES) can modulate cortical excitability through a plasticity-like mechanism and is considered to have therapeutic potentials in Parkinson’s disease (PD). However, the precise therapeutic value of such approach for PD remains unclear. Accordingly, we adopted a PD rat model to determine the therapeutic effects of CES. The current study was thus designed to identify the therapeutic potential of CES in PD rats.

**Methods:**

A hemiparkinsonian rat model, in which lesions were induced using unilateral injection of 6-hydroxydopamine (6-OHDA) into the medial forebrain bundle, was applied to identify the therapeutic effects of long-term (4-week) CES with intermittent theta-burst stimulation (iTBS) protocol (starting 24 h after PD lesion observation, 1 session/day, 5 days/week) on motor function and neuroprotection. After the CES intervention, detailed functional behavioral tests including gait analysis, akinesia, open-field locomotor activity, apomorphine-induced rotation as well as degeneration level of dopaminergic neurons were performed weekly up to postlesion week 4.

**Results:**

After the CES treatment, we found that the 4-week CES intervention ameliorated the motor deficits in gait pattern, akinesia, locomotor activity, and apomorphine-induced rotation. Immunohistochemistry and tyrosine hydroxylase staining analysis demonstrated that the number of dopamine neurons was significantly greater in the CES intervention group than in the sham treatment group.

**Conclusion:**

This study suggests that early and long-term CES intervention could reduce the aggravation of motor dysfunction and exert neuroprotective effects in a rat model of PD. Further, this preclinical model of CES may increase the scope for the potential use of CES and serve as a link between animal and PD human studies to further identify the therapeutic mechanism of CES for PD or other neurological disorders.

## Introduction

Parkinson’s disease (PD) is the second-most-frequently diagnosed neurodegenerative disease after Alzheimer’s disease and has become a major medical concern that affects 7–10 million individuals, close to 1% of the global population aged over 60 years (Nussbaum and Ellis, 2003; De Lau and Breteler, 2006; Wood-Kaczmar et al., 2006; Gilman, 2007; Kauffman et al., 2007; Reeve et al., 2014). The pathologic hallmark of the disease originates from degeneration of the dopaminergic neurons in the substantia nigra pars compacta (SNpc), which leads to several motor disturbances such as tremor, slowness, stiffness, balance problems, bradykinesia, akinesia, and gait disturbance (Rogers, 1996; Kalia and Lang, 2015; Moller et al., 2016; Sveinbjornsdottir, 2016). Mainstream modern PD treatment involves pharmacological approaches of dopamine supplementation (e.g., levodopa and l-3,4-dihydroxyphenylalanine) or dopamine agonist administration. Although dopaminergic drugs are effective treatments for controlling these symptoms in the initial stage of PD, associated complications such as levodopa-induced dyskinesia, freezing of gait, postural instability, depression, and motor fluctuations are common side effects after 5–10 years of levodopa administration, with the percentage of affected patients increasing over time (Obeso et al., 2000; Connolly and Lang, 2014; Bastide et al., 2015; Espay et al., 2018). Thus, it is still a high unmet need for developing the alternative and non-pharmacological therapeutic approach that can overcome the limitations of current treatment for PD.

Numerous alternative non-pharmacological and neuromodulatory approaches, such as repetitive transcranial magnetic stimulation (rTMS), transcranial direct current stimulation (tDCS), and cortical electrical stimulation (CES), are regarded as promising new therapeutic strategies for inducing the changes in neural activity and plasticity and considered as the new therapeutic strategies for PD (Pascual-Leone et al., 1994; Lefaucheur et al., 2004; Fasano et al., 2008; Elahi and Chen, 2009; Gutierrez et al., 2009; Degardin et al., 2012; De Rose et al., 2012). Among these techniques, compared with rTMS or tDCS, CES is a focused cortical stimulation technique that can provide higher focalization, spatial resolution and accuracy for stimulating a specific area of the motor cortex (Hsieh et al., 2015a). In clinical, CES has been used in neuropathic pain management (Son et al., 2006; Fagundes-Pereyra et al., 2010; Alm and Dreimanis, 2013). In addition, similar to the rTMS and tDCS, a recent animal study suggested that, CES can modulate motor cortical excitability through plasticity-like mechanisms (Hsieh et al., 2015a). Other preclinical studies have also demonstrated that CES can improve functional outcomes in ischemic stroke rats (Adkins-Muir and Jones, 2003; Adkins et al., 2006; Adkins et al., 2008). In human studies, it has been reported that CES of the motor cortex is not only effective in pain relief but also improves cognitive and motor functions in patients with advanced PD (Fasano et al., 2008; Gutierrez et al., 2009; De Rose et al., 2012). Although the CES approach has been reported in several human studies, studies that have applied CES as a long-term treatment in PD animals are few. In addition, several questions, such as what the optimal stimulation protocols and the underlying mechanisms of CES treatment for PD are, remain unclear (Cioni, 2007; Lefaucheur, 2009). Further experimental studies are required to determine the therapeutic value and the indications of CES in the treatment of PD.

For translational purposes, a diseased animal model could be the optimal means of studying the pathogenesis of PD. Such a model may provide a more stable condition, standardized stimulation protocol and controlled method of assessment to eliminate the discrepancies and clarify the treatment outcomes. To date, the detailed long-term effects of CES on PD-related symptoms as well as CES-induced neuroprotective effects have not been studied in PD animal models. Therefore, the current study was designed to identify the therapeutic potentials of CES in a neurotoxin-induced PD rat model. The therapeutic effects of CES were measured using behavioral assessments, including detailed time-course analysis of motor symptoms such as gait pattern, open-field locomotor activity, akinesia, drug-induced rotation and dopamine depletion levels. It was hypothesized that long-term CES intervention would reduce the aggravation of motor dysfunctions and have a neuroprotective effect on dopaminergic neurons in PD rat model. The knowledge obtained in the current study may have translational relevance for developing new therapeutic applications of CES in PD or other neurological disorders.

## Materials and Methods

### Animals

To avoid the hormonal influences on PD lesions (Gillies et al., 2014), experiments were conducted on 16 male Sprague Dawley rats (9 weeks old; body weight, 300–350 g) obtained from the BioLASCO Taiwan Co., Ltd., Taipei City, Taiwan. All rats were housed in a temperature-controlled animal care facility to a 12 h photoperiod (0700–1,900 h light) and temperature of 25°C with freely available food and water. All animal procedures were approved by the Institutional Animal Care and Use Committee at Chang Gung University. All efforts were made to minimize the number of rats required for the current study.

### Parkinson’s Disease Rat Model

In the current study, the classical model of intracerebral injection of neurotoxin 6-hydroxydopamine (6-OHDA) in rats was applied. The animal preparations and procedures for the induction of the PD rat model were in accordance with previous studies (Hsieh et al., 2011; Lee et al., 2012; Hsieh et al., 2015b; Feng et al., 2020). Briefly, rats were anesthetized using intraperitoneal injection of Zoletil 50 (50 mg/kg, i.p. Zoletil, Vibac, Carros, France) with xylazine (10 mg/kg, Rompun, Bayer, Barmen, Germany) and then mounted on a stereotactic apparatus (Model 940, David Kopf Instruments, Tujunga, CA, United States). A 2-cm midline incision was made along the scalp, and the implantation area was carefully cleared with H_2_O_2_ to expose the bregma line. A 6-hydroxydopamine solution (6-OHDA; 8 μg dissolved in 4 μl 0.02% ascorbic saline, Sigma-Aldrich, Burlington, MA, United States) was injected intracranially into the left medial forebrain bundle at a rate of 0.5 μL/min (AP: –4.3 mm; ML: + 1.6 mm; DV: –8.2 mm) by using a 10-μL microsyringe (84877, Hamilton, OH, United States) in accordance with the stereotaxic brain atlas of Paxinos and Watson (2005). The needle was maintained in the brain for 5 min before being slowly withdrawn to avoid backfilling along the injection tract. After inducing the PD lesion, we verified the effectiveness of PD lesion at 4 weeks post-surgery by using an apomorphine-induced rotational test. The rats that did not exhibit apomorphine-induced contralateral rotation behaviors were excluded for further analysis.

### Cortical Electrical Stimulation Electrode Implantation

**FIGURE 1:**
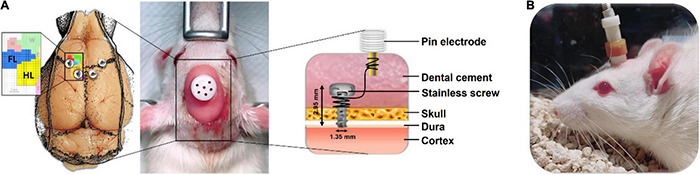
Placement and assembly details of the cortical electrical stimulation (CES) electrodes for the long-term treatment of CES. **(A)** The CES electrodes were positioned at the motor cortex of the forelimb and hindlimb and connected to the six-channel pedestal with wires and fixed with dental cement. **(B)** During the CES treatment, the head electrode pedestal served as the plugin site of the electrode pin to conduct electrical current.

After 6-OHDA injection, the CES electrode was then implanted on the rat’s skull under the same anesthesia. Stainless steel epidural screw electrodes (1.6-mm-diameter pole, 0–80 × 1/16, PlasticsOne Inc., Roanoke, VA, United States) were implanted into the four burr holes. Cortical electrodes were placed epidurally into the primary motor cortex of the forelimb (AP: −1.5 mm from the bregma; ML: ± 4.0 mm from the midline) and hind limb (AP: 1.0 mm from the bregma; ML: ± 1.25 mm from the midline) in accordance with functional rat brain mapping (Figure 1A; Fonoff et al., 2009). Four electrodes were inserted into a six-channel pedestal (MS363, Plastics One, Inc., Roanoke, VA, United States) by using wires. The screw electrodes and pedestal were secured to the skull surface with dental acrylic (Lang Dental Mfg., Wheeling, IL, United States). To focally stimulate the motor cortex of the rats, CES was applied from outside through an implantable plastic socket (E363/0 and MS363, Plastics One, Roanoke, VA, United States) (Figure 1B).

### Cortical Electrical Stimulation Treatment and Experimental Design

**FIGURE 2:**
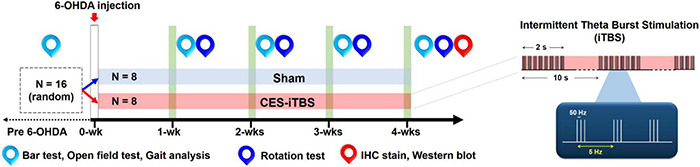
Experimental design for the long-term CES treatment. CES treatment was performed 7 days a week for 4 successive weeks. Behavioral tests, including bar test, open-field test, gait analysis, and apomorphine-induced rotation behavior, were performed weekly to investigate the treatment effects over time. Immunohistochemistry (IHC) and western blot analyses were applied on the week-4 post PD lesion to identify the neuroprotective effects of dopaminergic neurons and fibers following 4 weeks of CES treatment.

To verify the therapeutic effects of CES, the 6-OHDA-induced PD rats were randomly assigned to one of two groups, a sham CES treatment group (*n* = 8) or a CES treatment group (*n* = 8; Figure 2). In the CES treatment group, awake PD rats were treated with CES and an intermittent theta-burst stimulation (iTBS) protocol (triplets of pulses at 50 Hz, repeated every 200 ms with a 2-s sequence of TBS repeated every 10 s for 20 repetitions to a total of 600 pulses per session) (Huang et al., 2005). Based on our earlier study, the severity of dopamine depletion could be critically involved for the expression of motor plasticity. It might further interfere the improvement of motor performance (Hsieh et al., 2015b). Accordingly, for elucidating the possible therapeutic effects, we therefore conducted to have the early intervention of CES before the onset of symptoms. One day after a PD lesion, the PD animals in the CES treatment group received the CES protocol (1 session/day, 28 consecutive sessions over 4 weeks) (Figure 2). The intensity of electrical pulses was set at 80% resting motor threshold, which was defined as the minimal intensity of magnetic stimulation required to elicit minimal forelimb muscle twitches. In the sham CES treatment group, the PD rats also underwent the same CES protocol, but no electrical stimulation was applied at the same time points. All behavioral examinations were performed before and after treatment by a well-trained examiner who was blinded to treatment types. The timetable of behavioral and biochemistry analyses is summarized in Table 1. Behavioral tests, including the bar test for akinesia, open-field locomotor activity and gait analysis were conducted before as well as every week after the 6-OHDA lesions. Apomorphine-induced rotation was performed every week after PD lesion. Three motor behavior tests (bar test, open field test, gait analysis) were performed in same sequence on same day. The rotational test was conducted on alternate day after motor behavior tests. For each test, there were at least 3 h of resting time between each behavioral test. To identify the neuroprotective effect of CES treatment on dopaminergic neurons and fibers, immunohistochemistry and western blot analysis were conducted at week 4 after PD lesion.

**TABLE 1:** Timetable of behavioral and biochemical examinations on Parkinson’s disease (PD) rat model.

Assay	Weeks after 6-OHDA lesion					
	Pre 6-OHDA	0-wk	1-wk	2-wks	3-wks	4-wks
Bar test	**+**	**−**	**+**	**+**	**+**	**+**
Open field test	**+**	**−**	**+**	**+**	**+**	**+**
Gait analysis	**+**	**−**	**+**	**+**	**+**	**+**
Rotation test	**−**	**−**	**+**	**+**	**+**	**+**
IHC stain	**−**	**−**	**−**	**−**	**−**	**+**
Western blot	-	**−**	**−**	**−**	**−**	**+**

### Behavioral Test

The behavioral tests were measured at baseline and weekly for 4 weeks. The tests included bar test for akinesia, open-field locomotor activity, gait pattern, and apomorphine-induced rotation in the 6-OHDA lesioned rats with and without CES treatment.

### Akinesia

Akinesia is a cardinal motor symptom of PD. In previous studies, the bar test has been performed to observe the akinesia phenomenon in rat models of PD (Mabrouk et al., 2009; Hsieh et al., 2011). At the outset of the bar test, each rat was placed gently on a platform. Both forelimbs were placed at the same time on a horizontal acrylic bar (0.7 cm diameter), which was held 9 cm above the table surface. The total duration (in seconds) spent from placing of each forepaw on the bar to the first complete removal from the support bar was recorded (Hsieh et al., 2011; Feng et al., 2020; Hsieh et al., 2021). All PD rats were measured over five subsequent trials and the durations of these trials were averaged.

### Apomorphine-Induced Rotation Test

To verify the dopamine depletion levels after unilateral 6-OHDA infusion in PD rats, the conventional and reliable apomorphine-induced contralateral rotation test was performed (Creese et al., 1977; Iancu et al., 2005; Metz et al., 2005; Yoon et al., 2007; Hsieh et al., 2011). In brief, the rats were injected with apomorphine (0.5 mg/kg, dissolved in 0.02% ascorbic saline, Sigma-Aldrich, Burlington, MA, United States) and placed in a round bowl with a diameter of 40 cm. The number of rotations in a clockwise (contralateral) direction over 60 min was recorded on video and then manually counted using the trained examiner. Generally, a rotational response of over 300 rotations/h may suggest a maximum of 90% dopaminergic neuronal loss in the substantia nigra (Blandini et al., 2007; Hsieh et al., 2011). The rotation test was used to confirm the effectiveness of 6-OHDA infusion at 4 weeks post PD lesion. The rats that exhibited no contralateral rotation behavior induced by apomorphine were excluded from further analysis.

### Open-Field Locomotor Activity

The open-field test was commonly applied to measure general locomotor activity in 6-OHDA induced PD rat model (Silva et al., 2016; Su et al., 2018; Feng et al., 2020). In the open field test, each rat was monitored on video camera in an open-field black plexiglass arena (60 cm × 60 cm × 100 cm in dimension), as previously described (Yang et al., 2016; Feng et al., 2020). The total distance traveled and the movement time of each animal during a 10-min testing period was recorded on a digital camera (C930e, Logitech, Newark, CA, United States) and analyzed using a video-tracking system (Smart v3.0, Panlab SL, Barcelona, Spain). The testing arena was completely cleansed with 75% ethanol in the periods between each rat’s test session.

### Gait Analysis

To understand the motor behavioral changes in the PD rats before and after CES treatment, a detailed gait analysis was performed to assess gait disturbances in the PD rats. The procedure for measuring gait pattern was as described in previous works (Hsieh et al., 2011; Lee et al., 2012; Hsieh et al., 2021). In brief, the walking track equipment consisted of an enclosed walkway made of transparent plexiglass (80 cm × 6 cm × 12 cm length, width, and height, respectively) with a mirror (tilting at 45°) positioned underneath the walkway. A high-speed and high-resolution camera (PX-100, JVC, Yokohama, Japan) was positioned in the front of the walkway to capture a sagittal view and a downward view reflected by the mirror. After recording, we captured the image data from each trial and processed them semi-automatically by using the Matrix Laboratory software (MathWorks, version 9.6., R2019a) to identify the sequential footprints. Four spatial parameters (i.e., step length, stride length, base of support, and foot angle) and three temporal gait parameters (i.e., walking speed, stance/swing phase time, and stance/swing ratio) were determined (Hsieh et al., 2011; Lee et al., 2012; Liang et al., 2014).

### Histology Investigation

To assess the dopaminergic loss in the nigrostriatal neurons and fibers in PD rats, tyrosine-hydroxylase (TH) staining was performed (Hsieh et al., 2011; Li et al., 2015; Feng et al., 2020). The 6-OHDA-lesioned rats from both groups were sacrificed after behavioral tests at postlesion week 4. The procedure for the TH staining was as described previously (Truong et al., 2006; Hsieh et al., 2011; Hsieh et al., 2015b; Feng et al., 2020). In brief, the rat brains were carefully removed, postfixed with 4% paraformaldehyde fixative solution (PFA), and then placed under 48-h cryoprotection at 4°C with 30% sucrose solution until the brain sank. The cerebral tissues were sectioned into 30-μm-thick coronal blocks by using a cryostat (Leica CM3050 S Cryostat, Miami, FL, United States). The substantia nigra pars compacta (SNpc) and striatum areas were selected. The free-floating sections were quenched for 10 min with 0.3% H_2_O_2_–phosphate-buffered-saline (PBS) and blocked non-specific antibodies for 1 h with 10% milk. The sections were then incubated with anti-TH rabbit primary antibody (1:1,000, AB152, Millipore, Burlington, MA, United States) at room temperature for 1 h. Thereafter, the sections were washed three times with PBS, incubated with antirabbit secondary antibody (1:200, MP-7401, Vector Labs, Burlingame, CA, United States) at room temperature for 1 h, and placed in 3,3-diaminobenzidine (DAB, SK-4105, Vector Labs, Burlingame, CA, United States) solution for 3–5 min. Finally, the sections were mounted on slides and treated with graded alcohols to dehydrate them. To remove excess dyes and make the samples clear for microscopic observation, the brain tissue sections were cleaned using xylol (Sinopharm, Beijing, China) before being coverslipped in dibutyl phthalate polystyrene xylene. Three brain slices were collected and used for further analysis in each rat. The sections were digitally scanned by using a digital pathology slide scanner (Aperio CS2, Leica Biosystems Inc., Buffalo Grove, IL, United States). The obtained digital images were then converted into binary (8-bit black-and-white) images. In each slide, two fields, including SNpc and striatum in the non-lesioned and lesioned areas were chosen for further analysis. The numbers of TH-positive cells in the SNpc were counted by means of particle analysis using Image-Pro Plus 6.0 software (Media Cybernetics, Bethesda, MD, United States) and these values were then manually validated by two investigators to ensure the correct identification of TH-positive pattern (Hsieh et al., 2021). The percentage of TH-positive cells was calculated in the ipsilateral hemisphere and normalized with respect to the contralateral side (Feng et al., 2020; Hsieh et al., 2021). With regard to the striatal TH-positive fibers, the same image analysis software (Image-Pro Plus 6.0, Media Cybernetics, Bethesda, MD, United States) was used to correct the non-specific background density measured at the corpus callosum, and the optical density of the TH-positive fibers in the striatum of each hemisphere was analyzed separately (Hsieh et al., 2011; Feng et al., 2020; Hsieh et al., 2021). The percentage of TH-positive fibers on the ipsilateral side was normalized and presented with respect to the contralateral side.

### Western Blot

Approximately 4 μg of tissue-lysate proteins was fractionated and separated using 12% sodium dodecyl sulfate-polyacrylamide gel and then blotted onto a polyvinylidene difluoride membrane (Millipore, Burlington, MA, United States). To prevent antibodies from binding to the membrane non-specifically, the membrane was blocked with 5% skim milk diluted in Tris-buffered saline with Tween 20 buffer. Dopaminergic neuron levels were calculated using a blotting membrane with indicated primary antibodies, including anti-TH (1:100, Merck, Darmstadt, Germany) and mouse anti-β-actin (1:100, Santa Cruz Biotechnology, Santa Cruz, CA, United States). After incubation with anti-mouse IgG antibodies (enzyme horseradish peroxidase [HRP]; 1:5,000, GeneTex, Irvine, CA, United States) for 1 h at room temperature, the protein intensity was determined using a chemiluminescent HRP substrate (Millipore, Burlington, MA, United States). The bands were digitally scanned, and the intensities were quantified using Image J software (National Institutes of Health, Bethesda, MD, United States).

### Data Analysis

Statistical analysis was performed using the SPSS 25.0 package (IBMCorp., Armonk, NY, United States). The significance level was set at *p* < 0.05. The effect of CES treatment for all data measures was assessed using two-way repeated-measures analysis of variance (ANOVA) with protocol (sham vs. CES treatment) used as the between-subjects factor and time (pre-assessment vs. post-assessment) as the within-subject factor. Unpaired *t* tests were performed to compare groups at each time point when the main effect of the group was significant. Furthermore, separate one-way ANOVAs, followed by *post-hoc* Bonferroni tests, were used to compare behavioral, immunohistochemistry and Western blot data at distinct time points as necessary.

## Results

### Effects of Cortical Electrical Stimulation Treatment on Akinesia After 6-Hydroxydopamine Lesion

**FIGURE 3:**
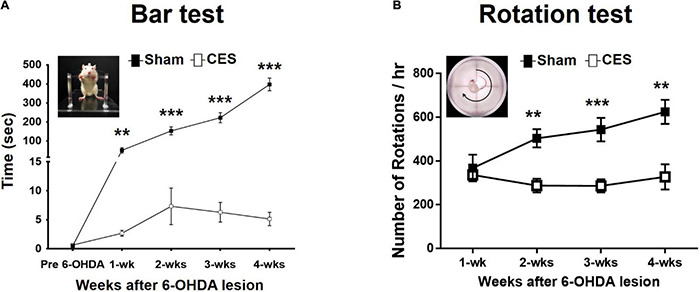
Time-course analysis of bar test **(A)** and apomorphine-induced rotational behavior **(B)** in the sham-CES-treated and CES-treated groups after the 6-hydroxydopamine (6-OHDA) lesions. Values are shown as means ± SEMs. *p < 0.05, **p < 0.01, and ***p < 0.001; each indicates significant difference between the two groups.

All behavioral tests were completed in eight PD-lesioned rats that underwent 4 weeks of sham treatment and in another eight such rats that underwent 4 weeks of CES treatment. The akinesia phenomenon was observed using the prelesion and post-6-OHDA-lesion week 1–4 using bar test. Repeated-measures ANOVA revealed significant differences main effects of time (*F*_(4,56)_ = 62.483, *p* < 0.001), group (*F*_(1,14)_ = 148.764, *p* < 0.001), and time × group interaction (*F*_(4,56)_ = 59.823, *p* < 0.001). Moreover, the subsequent *post-hoc t*-tests revealed that the duration in the bar test reached significant differences between the two groups at 1 week (*t* = 3.406; *p* = 0.004), 2 weeks (*t* = 6.875; *p* < 0.001), 3 weeks (*t* = 8.084; *p* < 0.001), and 4 weeks (*t* = 11.866; *p* < 0.001) after the 6-OHDA lesions were observed (Figure 3A).

### Effects of Cortical Electrical Stimulation Treatment on Apomorphine-Induced Rotation

In this study, we observed the contralateral rotations by using apomorphine-induced rotation tests from postlesion weeks 1–4 to characterize the extent of motor impairment behavior of PD rats with the disease progression. All animals exhibited the apomorphine-induced contralateral rotation behavior at 4 weeks post PD lesion. Therefore, all 16 rats (8 rats for sham CES treatment group and 8 rats for CES treatment group) were included for data analysis. Repeated-measures ANOVA revealed significant differences main effects of time (*F*_(3,42)_ = 5.162, *p* = 0.004), group (*F*_(1,14)_ = 14.757, *p* = 0.002), and time × group interaction (*F*_(3,42)_ = 6.678, *p* = 0.001). Moreover, according to the results of the *post-hoc t*-tests at each time point, the differences were mainly influenced by CES treatment effects, which was chiefly observed in the number of rotations per hour at 2 weeks (*t* = 4.146; *p* = 0.001), 3 weeks (*t* = 4.194; *p* = 0.001), and 4 weeks (*t* = 3.735; *p* = 0.002) after 6-OHDA lesion (Figure 3B).

### Effects of Cortical Electrical Stimulation Treatment on Open-Field Test Locomotor Activity Levels

**FIGURE 4:**
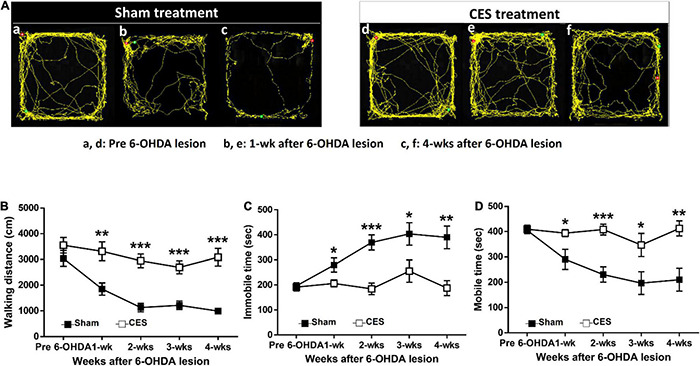
Time-course analysis of locomotor activity assessed by open-field test at before and after 6-OHDA lesions. **(A)** Representative traces of rat movement patterns during the open-field test in the sham- and CES-treaded rats before PD lesions and at postlesion weeks 1 and 4. **(B)** Significant differences were observed between the sham and CES treatment groups in walking distance, **(C)** immobile time, and **(D)** mobile time after PD lesions. Values are expressed as the means ± SEMs. *p < 0.05, **p < 0.01, and ***p < 0.001; each indicates a significant difference between the two groups.

The open-field test is a common behavioral test used to evaluate the locomotor activity. The measured outcomes, including walking distance, immobile time and mobile time, were used to investigate the differences in general locomotor activity between the sham and CES treatment groups from the prelesion stage and throughout postlesion weeks 1–4. In the open field test, the overall traveled walking distance between the CES-treated rats and sham-CES treated rats following 6-OHDA PD lesion was calculated (Figure 4A). A repeated-measures ANOVA revealed significant differences main effects of time (*F*_(4,56)_ = 15.872, *p* < 0.001), group (*F*_(1,14)_ = 29.822, *p* < 0.001), and time × group interaction (*F*_(4,56)_ = 4.325, *p* = 0.004). Moreover, according to the *post-hoc t*-test results, the differences were mainly influenced by CES treatment effects across every time point, primarily for walking distance at postlesion weeks 1 (*t* = –3.415; *p* = 0.004), 2 (*t* = –5.831; *p* < 0.001), 3 (*t* = –4.575; *p* < 0.001), and 4 (*t* = –5.751; *p* < 0.001; Figure 4B). With regard to the immobile time, repeated-measures ANOVA revealed significant differences main effects of time (*F*_(4,56)_ = 9.608, *p* < 0.001), group (*F*_(1,14)_ = 14.338, *p* = 0.002), and time × group interaction (*F*_(4,56)_ = 5.851, *p* = 0.001). Moreover, according to the *post-hoc t*-test results, the differences were mainly driven by CES treatment effects across every time point, primarily for immobile time at postlesion weeks 1 (*t* = –2.610; *p* = 0.021), 2 (*t* = –4.857; *p* < 0.001), 3 (*t* = –2.321; *p* = 0.036), and 4 (*t* = –3.727; *p* = 0.002; Figure 4C). With regard to the mobile time, repeated-measures ANOVA revealed significant differences main effects of time (*F*_(4,56)_ = 9.539, *p* < 0.001), group (*F*_(1,14)_ = 14.386, *p* = 0.002), and time × group interaction (*F*_(4,56)_ = 5.835, *p* = 0.001). Furthermore, according to *post-hoc t*-test results, the differences were mainly influenced by CES treatment effects across every time point, primarily in mobile time at postlesion weeks 1 (*t* = 2.609; *p* = 0.021), 2 (*t* = 4.858; *p* < 0.001), 3 (*t* = 2.327; *p* = 0.036), and 4 (*t* = 3.727; *p* = 0.002; Figure 4D).

### Effects of Cortical Electrical Stimulation Treatment on Gait Pattern

**FIGURE 5:**
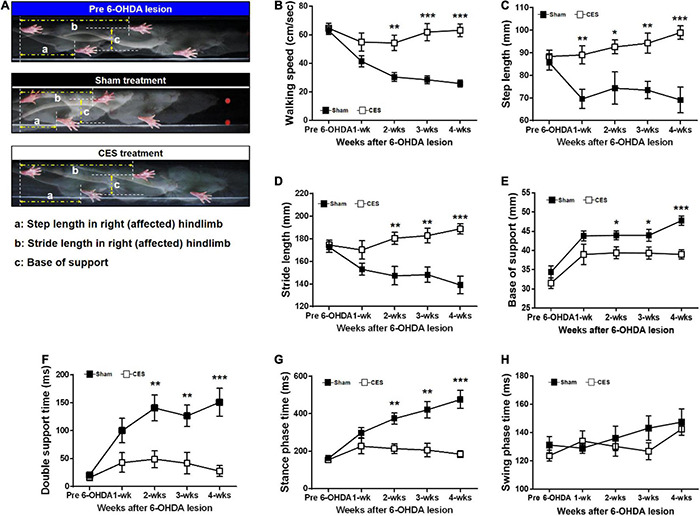
**(A)** Characteristics of stepping of rat (as indicated by footprints) during walkway locomotion prior to PD lesion and postlesion week 4 for a sham treatment group rat and a CES-treated rat. Time-course changes in walking speed **(B)**, step length **(C)**, stride length **(D)**, base of support **(E)**, double support time **(F)**, stance phase time **(G)**, and swing phase time **(H)** in the sham-treated and CES-treated PD rats over the 4 weeks of observation. Notably, the walking speed, step, and stride lengths decreased significantly in the sham CES treatment group but were less affected in the CES treatment group. Gradual increases in the base of support were found in both groups, but the CES treatment group exhibited a lower increase than the sham treatment group. *p < 0.05, **p < 0.01, and ***p < 0.001, each of which indicated significant differences between two groups at each time point (unpaired *t*-tests).

In this study, a quantitative analysis of gait pattern in PD rats with and without CES treatment was performed. Figure 5A presents typical footprint images captured from the PD rats that underwent sham and CES treatments before PD lesion and at postlesion week 4. Repeated-measures ANOVA on gait parameters revealed significant time × group interaction in walking speed (*F*_(4,56)_ = 15.80, *p* < 0.001), step length (*F*_(4,56)_ = 4.51, *p* = 0.003), stride length (*F*_(4,56)_ = 6.04, *p* < 0.001), double support time (*F*_(4,56)_ = 4.756, *p* = 0.002), stance phase time (*F*_(4,56)_ = 10.488, *p* < 0.001), suggesting less impairment of gait pattern in the CES treatment group than in the sham CES treated group (Figures 5B–D,F,G). No significant group × time interaction was found in base of support (*F*_(4,56)_ = 0.65, *p* = 0.632) and swing phase time (*F*_(4,56)_ = 0.901, *p* = 0.47) (Figures 5E,H). *Post-hoc t*-tests between the two groups showed that walking speed, step length, stride length, base of support, double support time and stance phase time reached a significant difference at 2, 3 and 4 weeks after PD lesion (Figures 5B–G).

**FIGURE 6:**
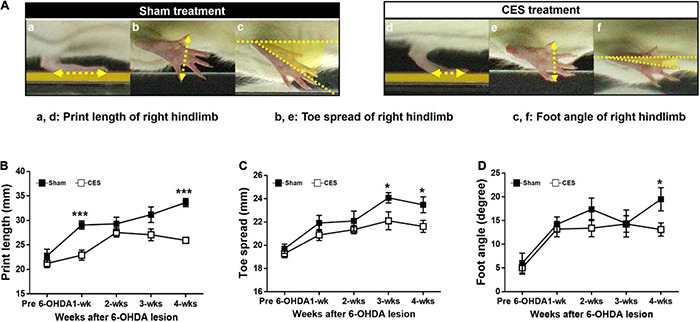
Characteristics of footprint during walkway locomotion in PD rats with sham-treatment or CES treatment after 4 weeks PD lesion **(A)**. Time-course changes in print length **(B)**, toe spread **(C)**, and foot angle **(D)** in the sham- and CES-treated PD rats over 4 weeks of observation. Note that the print length, toe spread, and foot angle increased in the sham CES treatment group but less affected in the CES treatment group. *p < 0.05, **p < 0.01, and ***p < 0.001, significant differences between two groups at each time point (unpaired *t*-tests).

Figure 6A shows footprint images captured from PD rats with sham and CES treatment at week 4 following PD lesion. Repeated measures ANOVA on foot parameters during locomotion revealed significant main effect of group in print length (*F*_(1,14)_ = 17.02, *p* = 0.001), toe spread (*F*_(1,14)_ = 6.426, *p* = 0.024) but not in foot angle (*F*_(1,14)_ = 1.254, *p* = 0.282). The differences were mainly driven by CES treatment according to the results of *post-hoc t*-test at each time point, which chiefly observed in print length and toe spread stride at the 4th week of 6-OHDA lesion (unpaired *t*-tests, *p* < 0.05) (Figures 6B–D).

### Quantification of Dopaminergic Neuronal Degeneration Levels

**FIGURE 7:**
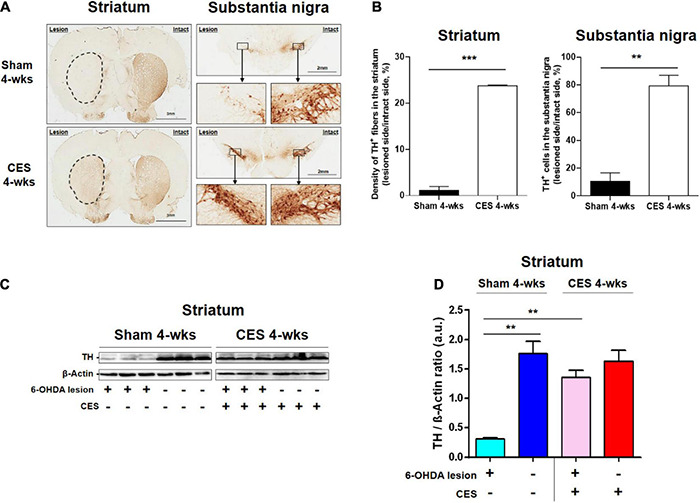
**(A)** Representative TH-positive fibers in the striatum and TH-positive neurons in the substantia nigra pars compacta from the sham-treated and CES-treated rats at 4 weeks after PD lesion. **(B)** Comparison of the average TH-positive fibers and neurons between sham and CES treatment groups. **(C)** Expression of TH in the striatum represented through a western blot for PD rats that received sham (*n* = 3) and CES (*n* = 3) treatments at 4 weeks after PD lesion. **(D)** Quantitative analysis of relative protein levels of TH in the striatum in the sham and CES treatment groups. **p < 0.01, ***p < 0.001, each of which indicates a significant difference between the two groups.

To observe the effects of 4-week CES treatment on dopaminergic neurons and fibers in the nigrostriatal pathway, we measured TH-positive cells in the substantia nigra and TH-positive fibers in the striatum by using immunohistochemistry staining. The results of the TH immunohistochemistry in the substantia nigra and striatum in PD rats that received and did not receive CES treatment for the 4 weeks are illustrated in Figure 7A. The quantification of TH-positive fiber loss in the striatum and TH-positive cell loss in the substantia nigra at 4 weeks post PD lesion are presented in Figure 7B. PD rats that received CES treatment exhibited a greater preservation of TH-positive fibers in the striatum (*t* = 26.879, *p* < 0.001) and TH-positive cells in the substantia nigra (*t* = 7.055, *p* = 0.002) compared with sham treatment group.

Furthermore, we examined the expression levels of TH protein of the lesioned striatum in the sham or CES treatment group through western blotting. The TH levels in the striatum are illustrated in Figure 7C. Compared with the CES treatment group, the sham treatment group exhibited lower TH levels in the striatum. *Post-hoc* test in TH levels showed significant difference between the sham-treated and CES-treated PD rats (*t* = 8.576; *p* < 0.01; Figure 7D).

## Discussion

In the current study, we evaluated the effects of CES treatment in a 6-OHDA induced PD rat model. We performed several comprehensive quantitative assessments under various aspects of motor function (all of which are commonly affected in PD patients) to identify the therapeutic effects over the 4 weeks of CES treatment. We found that such a long-term CES treatment with iTBS protocol could mitigate 6-OHDA-induced motor dysfunction and potentially exert a neuroprotective effect on dopaminergic neurons and fibers. After 4 weeks of CES intervention, several types of motor behavioral impairment were reduced. In addition, the histological results revealed greater preservation of dopaminergic neurons and striatal fibers in the CES treated group than in the sham-treated group, a finding that was verified through western blotting, further indicating the neuroprotective effect of CES. To the best of our knowledge, this is the first study to comprehensively investigate the therapeutic effects of CES on several aspects of motor function in a PD rat model. These data could enhance the growing body of basic and clinical research on the efficacy of CES in PD treatment.

In this study, CES with iTBS protocol was applied to induce changes in long-term potentiation and plasticity in disease progression. The pattern of neurodegeneration in PD involves abnormal neuronal activities in the basal ganglia–thalamo–cortical circuits. For example, the usual effect of the facilitation of thalamic projections to the motor cortex is reduced in PD, resulting in deactivation or hypoactivation of cortical motor areas, and thus, reduced motor output during movement (Wichmann and Delong, 1996; Lefaucheur, 2005; Pasquereau et al., 2015). Functional neuroimaging studies have also indicated that the motor cortex is impaired in both the early and late stages of PD. For example, hypoactivation in the primary motor cortex seems to be observed preferentially by functional magnetic resonance imaging (fMRI) in early, untreated patients (Ellaway et al., 1995; Buhmann et al., 2003). By contrast, hyperactivity of the motor cortex has more often been found in patients with more advanced PD (Sabatini et al., 2000; Haslinger et al., 2001). It indicates that the motor cortex may be attributed to a compensatory cortical reorganization due to the disease progression (Rascol et al., 1998). The available anatomical, physiological, and imaging-based studies suggest that PD results from dopamine loss in the basal ganglia, which induces neuronal discharge abnormalities within the entire motor circuit (Delong and Wichmann, 2007). In addition to the basal ganglia pathway, at the cortical level, this manifests in the form of abnormalities in cortical activation patterns during movement tasks (Delong and Wichmann, 2007). Thus, in addition to the basal ganglia circuit, targeting the motor circuitry in neuromodulation approaches can be also be considered as a therapeutic strategy for PD. Previous research has demonstrated that both long-term potentiation and depression-like plasticity of the motor cortex are impaired in individuals with PD (Kishore et al., 2011; Suppa et al., 2011). On the basis of histological and behavioral investigations, our earlier study suggested that such impaired motor plasticity is strongly associated with the loss of dopaminergic nigral cells and striatal fibers (Hsieh et al., 2015b). Thus, in the present study, the early intervention of brain stimulation was designed to induce superior neuroprotective effects and CES-induced motor functional changes in the PD rats.

Until now, the therapeutic effects and potential mechanisms of CES in the treatment of PD have remained unclear. The animal models may help provide greater insight into these mechanisms. In the histological results of IHC staining in the two groups, CES treatment could effectively reduce the degeneration level of TH-positive fibers and TH-positive cells. We also found that 6-OHDA-lesioned rats treated with CES for 4 weeks registered reductions in akinesia and gait disturbances. These results are consistent with the findings of a human study that demonstrated the effectiveness of CES in improving cognitive and motor function in patients with advanced PD (Fasano et al., 2008; Gutierrez et al., 2009; De Rose et al., 2012). Although the optimal stimulation parameters of CES remain unclear, a suitable animal model of the disease could help identify an effective stimulation protocol, including adjustment and optimization of frequency, polarity, and current level, allowing rapid screening and neurophysiological analysis in PD animal studies. Future research is still required to clarify the mechanisms underlying the effects of CES and explore and optimize CES protocols in PD.

Moreover, the western blot results verified the TH protein levels, indicating that long-term CES intervention might not only ameliorate the deterioration of motor behavior caused by 6-OHDA lesions but also promote the retention of more dopaminergic neurons under the threat of neurotoxin. Most motor functions were significantly well preserved after 3–4 weeks of CES treatment. Based our previous study, after 24 h of the 6-OHDA injection, the degeneration of dopaminergic neurons and striatum terminal was observed (Hsieh et al., 2011). Hence, combined with the histological and behavioral results, this study suggests that early (starting from directly after the appearance of PD lesions) and long-term (4-week) CES intervention may help mitigate the threat of neurodegeneration, maintain motor functions and reduce dopaminergic neuron death. Similar to our findings, earlier studies reported that rTMS could induce neuroprotective effects in stroke and brain injury animal models (Ljubisavljevic et al., 2015; Sasso et al., 2016). The neuroprotective effects on dopaminergic cells or fibers could be related to anti-inflammatory effects (e.g., tumor necrosis factor-alpha, TNF-α or cyclooxygenase-2, COX-2), upregulation of neurotrophic factor (e.g., brain-derived neurotrophic factor, BDNF; glial cell line-derived neurotrophic factor, GDNF) or reduction of astrogliosis and microglial activation (glial fibrillary acidic protein, GFAP; ionized calcium binding adaptor molecule 1, Iba-1) induced by long-term CES intervention (Yang et al., 2010; Lee et al., 2013; Sasso et al., 2016; Cacace et al., 2017). Because the exact mechanism underlying these effects remains unclear, further investigation is required to clarify these potential mechanisms underlying the neuroprotective effects of CES in PD.

## Conclusion

This study provided a clearer picture of the progressive changes in PD-related symptoms with or without CES treatment and documented the efficacy of CES in ameliorating motor dysfunctions and inducing the neuroprotective effect of the dopaminergic system in a 6-OHDA-induced PD rat model. The long-term CES treatment model used in this study may help bridge the gap between animal and human studies of PD. Future research is still required to further clarify the underlying mechanisms, allowing improved CES protocols and more effective therapies in PD and other neurological diseases to be developed for humans.

## Data Availability Statement

The raw data supporting the conclusions of this article will be made available by the authors, without undue reservation.

## Ethics Statement

The animal study was reviewed and approved by the Institutional Animal Care and Use Committee, Chang Gung University IACUC Approval No: CGU15-151, Period of Protocol: valid from August 01, 2016 to July 30, 2019.

## Author Contributions

C-WK, C-WP, and T-HH conceived and designed the experiments. C-WK, H-CT, P-KC, T-YJ, and T-HH performed the experiments. T-HH, M-YuC, C-YP, and C-WP provided the equipment. C-YP, C-WP, X-KH, H-HL, and T-HH developed the methodology. C-WK, H-HL, X-KH, H-CT, and T-HH analyzed the data. C-WK, M-YuC, M-YiC, X-KH, H-HL, TN, and T-HH contributed to the writing and editing the manuscript. All authors contributed to the article and approved the submitted version.

## Conflict of Interest

The authors declare that the research was conducted in the absence of any commercial or financial relationships that could be construed as a potential conflict of interest.

## Publisher’s Note

All claims expressed in this article are solely those of the authors and do not necessarily represent those of their affiliated organizations, or those of the publisher, the editors and the reviewers. Any product that may be evaluated in this article, or claim that may be made by its manufacturer, is not guaranteed or endorsed by the publisher.
